# Thioredoxin 1 (Trx1) is associated with poor prognosis in clear cell renal cell carcinoma (ccRCC): an example for the crucial role of redox signaling in ccRCC

**DOI:** 10.1007/s00345-021-03900-5

**Published:** 2021-12-02

**Authors:** Silvia Ribback, Stefan Winter, Tobias Klatte, Elke Schaeffeler, Manuela Gellert, Viktoria Stühler, Marcus Scharpf, Jens Bedke, Martin Burchardt, Matthias Schwab, Christopher H. Lillig, Nils Kroeger

**Affiliations:** 1 Institute of Pathology, University Medicine, Greifswald, Germany; 2 Dr. Margarete Fischer-Bosch Institute of Clinical Pharmacology, Stuttgart, University of Tübingen, Tübingen, Germany; 3 Department of Urology, Royal Bournemouth Hospital, Bournemouth, UK; 4 Institute of Medical Biochemistry and Molecular Biology, University Medicine, Greifswald, Germany; 5 Department of Urology, University of Tübingen, Tübingen, Germany; 6 Institute of Pathology, University of Tübingen, Tübingen, Germany; 7 Department of Urology, University Medicine, Ferdinand-Sauerbruch-Straße, E17475 Greifswald, Germany; 8 Departments of Clinical Pharmacology, Pharmacy and Biochemistry, University of Tübingen, Tübingen, Germany

**Keywords:** Thioredoxin, Oxidative signaling, Reactive Oxygen Species, Kidney cancer, Targeted Therapies

## Abstract

**Purpose:**

Thioredoxins are major regulatory proteins of oxidative signaling. Trx1 is the most prominent thioredoxin and, therefore, the current study sought to evaluate the prognostic role of Trx1 in ccRCC.

**Methods and patients:**

A tissue micro-array (TMA) study was carried out to evaluate the association of Trx1 with clinicopathological features and survival outcome. Data from the Cancer Genome Atlas (TCGA) were evaluated for the association of characteristics in the Trx1 gene with clinicopathological features and survival outcome.

**Results:**

In the TMA, patients with ccRCC that had high Trx1 levels had lower T stages (*p* < 0.001), less often distant metastases (*p* = 0.018), lower nuclear grades (*p* < 0.001), and less often tumor necrosis (*p* = 0.037) or sarcomatoid features (*p* = 0.008). Patients with a combined score of  ≥ 10 had better DSS than patients with a low combined score of < 10 (HR 95% CI 0.62 (0.39–0.98)). Interestingly, the survival outcome is compartment specific: ccRCC patients whose tumors had exclusively Trx1 expression in the cytoplasm had the worst survival outcome (HR 3.1; 95% CI 1.2–8.0). Genomic data from the TCGA demonstrated that patients with ccRCCs that had Trx1 losses had more advanced clinicopathological features and worse survival outcome in disease specific (*p* < 0.001), overall (*p* = 0.001), and progression free survival (*p* = 0.001) when compared to patients with ccRCCs without copy number variations (CNV) or gains.

**Conclusion:**

The current study suggests a possible role of Trx1 in the tumor biology of ccRCC and thus, the current study strongly advises in depth investigations of redox signaling pathways in ccRCC.

**Supplementary Information:**

The online version contains supplementary material available at 10.1007/s00345-021-03900-5.

## Introduction

Oxidative stress has been associated with carcinogenesis for many decades. An imbalance of pro-oxidants and antioxidants has been attributed to desoxyribonucleic acid (DNA) damage and failure in pivotal cellular processes, including metabolic deregulations, cellular membrane injuries and failure in DNA transcription and mRNA translation [1]. The thioredoxin family has formerly been considered as a major regulator of an oxidative equilibrium in cells. Thioredoxins are highly conserved throughout almost all cell species. They comprise three groups of proteins: the name giving thioredoxins (Trxs), glutaredoxins (Grxs), and peroxiredoxins (Prxs) (reviewed in [1]).

An overwhelming body of scientific literature has provided evidence that thioredoxins are not guardians of an oxidative equilibrium but better characterized as regulators of oxidative signaling (reviewed in [1, 2]). In this form, members of the thioredoxin family have functions as sensors, transducers, and effectors of cellular functions [3]. Biological processes such as activation and compartment stabilization of proteins cannot be explained without consideration of electrostatic characteristics [4]. In fact, it has been demonstrated that negative electric fields reaching into the solvent outside of the active sides of thioredoxins, electrostatic and geometry complementary determine substrate specify of thioredoxins and probably other proteins [4].

Trx1 has been involved in many biological processes that have a key role in tumorigenesis of clear cell renal cell carcinoma (ccRCC). For example, cytosolic Trx1 can be secreted and act as a chemokine that can attract and activate immune cells [1]. Furthermore, Trx1 can induce the expression of interleukin (IL) IL-1, IL-2, IL-8, and dose dependent the expression of IL-6 [1]. Particularly, IL-6 is a major regulatory protein of tumorigenesis in ccRCC [5]. Moreover, the macroscopic view of ccRCC tumors usually demonstrates a mixed picture of fatty, necrotic and hemorrhagic areas, which highlights that biological processes such as hyperactive metabolic pathways and neovascularization are key components for the development and progression of ccRCCs. Multiple studies have shown that members of the thioredoxin family are key regulatory components of cellular metabolism and also of neovascularization [1]. For example, serum levels of Trx1 have been correlated with diabetes susceptibility and with glucose tolerance. Additionally, Trx1 is able to induce the expression of vascular endothelial factor (VEGF) [1]. VEGF inhibition with tyrosine kinase inhibitors (TKIs) is one of the most important therapeutic strategies in the medical treatment of metastatic ccRCCs [6].

Finally, it has been shown that the hypoxia-inducible factor-2α (HIF-2α) is an important tumor progressor in ccRCC [7]. Trx1 regulates HIF-2α levels via an iron responsive element in vitro [8]. It has been demonstrated that depletion of Trx1 results in an increase of HIF-2α mRNA levels. The authors have suggested that HIF-2α accumulation requires either sufficient iron supply or the lack of the Trx1 reducing system. Currently, phase III clinical trials investigate HIF-2α inhibition as one of the future promising targets in mRCC therapy. Trx1 and the thioredoxin family may, therefore, be involved in ccRCC progression via a HIF-2α-dependent mechanism.

Collectively, there exists plausible evidence that Trx1 can induce pathophysiological processes that guide tumor progression in ccRCC. On the other hand, also the loss of Trx1 is may be able to induce HIF-2α accumulation. Low or increased levels of Trx1 may impact context depended on tumor progression in ccRCC. Therefore, the current study has investigated the association of Trx1 protein levels in a TMA analysis and it was hypothesized that both low or high levels of Trx1 could be associated with clinicopathological features and survival outcome of patients with ccRCC.

## Methods and patients

### Patient and tumor characteristics

Two patient cohorts were analyzed. The first cohort included patients who have been surgically treated at the Department of Urology at the University Hospital in Tübingen/Germany and were analyzed in a tissue micro-array (TMA) study (supplementary Table 1). All included patients had pathologically evaluated T stages. Lymphadenektomy was done when suspect lymph nodes were visible in pre-operative MRI or CT scans. Therefore, the T stage is was clinically and pathologically evaluated. The M stage was clinically evaluated with MRI and CT scans. All M1 patients were synchronically metastasized, and nephrectomy was performed in a cytoreductive intend in these patients. The second cohort included patients from The Cancer Genome Atlas project (TCGA) (suppl. Table 1).

### Tissue micro-array (TMA) analyses

Trx1 is known to be in both the nucleus and the cytoplasm. In both compartments, Trx1 is involved in redox and iron regulatory processes (reviewed in [1]). The staining was evaluated by an experienced genitourinary pathologist (S.R.). The intensity was counted on a 0–3 scale (0 = negative, 1 = weakly positive, 2 = moderately positive, 3 = strongly positive), and percentage of positively stained target cells (range 0–100% positive) staining at each intensity. To better represent overall protein levels, we combined the frequency and intensity measures into an integrated intensity measure using the following formula: ((% staining at intensity 3*3) + (% staining at intensity 2*2) + (% staining at intensity 1*1))/100 as described previously [10].

Trx1 protein data were correlated with disease-specific survival (DSS), clinicopathological features, and expression of marker proteins of pathways that are relevant to RCC biology. Median follow-up was 90.4 months (supplementary Table 1). Descriptive statistics included continuous variables that are shown as mean ± standard deviation (SD) or interquartile ranges (IQR), whereas categorical data are shown as absolute numbers and corresponding frequencies. Categorical variables were compared with the chi-square or Fisher’s exact test as appropriate. Either Student’s *t* test corrected with Levan’s test for equivalence or the Mann–Whitney *U* test were used for comparison of continuous variables. Cutoff values were calculated with recursive partition tree analyses using the rpart function of R. Survival functions were estimated with the Kaplan–Meier method and associations with survival times were assessed with uni- and multivariable Cox proportional hazard (PH) regression analyses. All statistical tests were two-sided and statistical significance was defined as *p* < 0.05. All data were analyzed with the Statistical Package for Social Sciences software, version 24.0 (SPSS Inc., Chicago, Il) and R version Rx64 3.5.0 (www.r-project.org), including additional packages coin, survival and rms.

### Analysis of genetic alterations in the TCGA

Associations between clinicopathological variables and copy number variations (CNV) or somatic mutations were investigated using Fisher’s exact test, Cochran-Armitage trend test or Mann–Whitney *U* test as appropriate. Uni- and multivariable Cox PH regression were applied for association analyses between CNV or somatic mutations and overall survival (OS), disease-specific survival (DSS), and progression free interval (PFI). Survival data were obtained from Liu et al*.* [12]. Restricted cubic splines were used to model the relationship between Trx1 RNA levels and the survival endpoints OS, DSS, and PFI (Figure S4).

## Results

### Results of the tissue micro-array analysis.

**Fig. 1:**
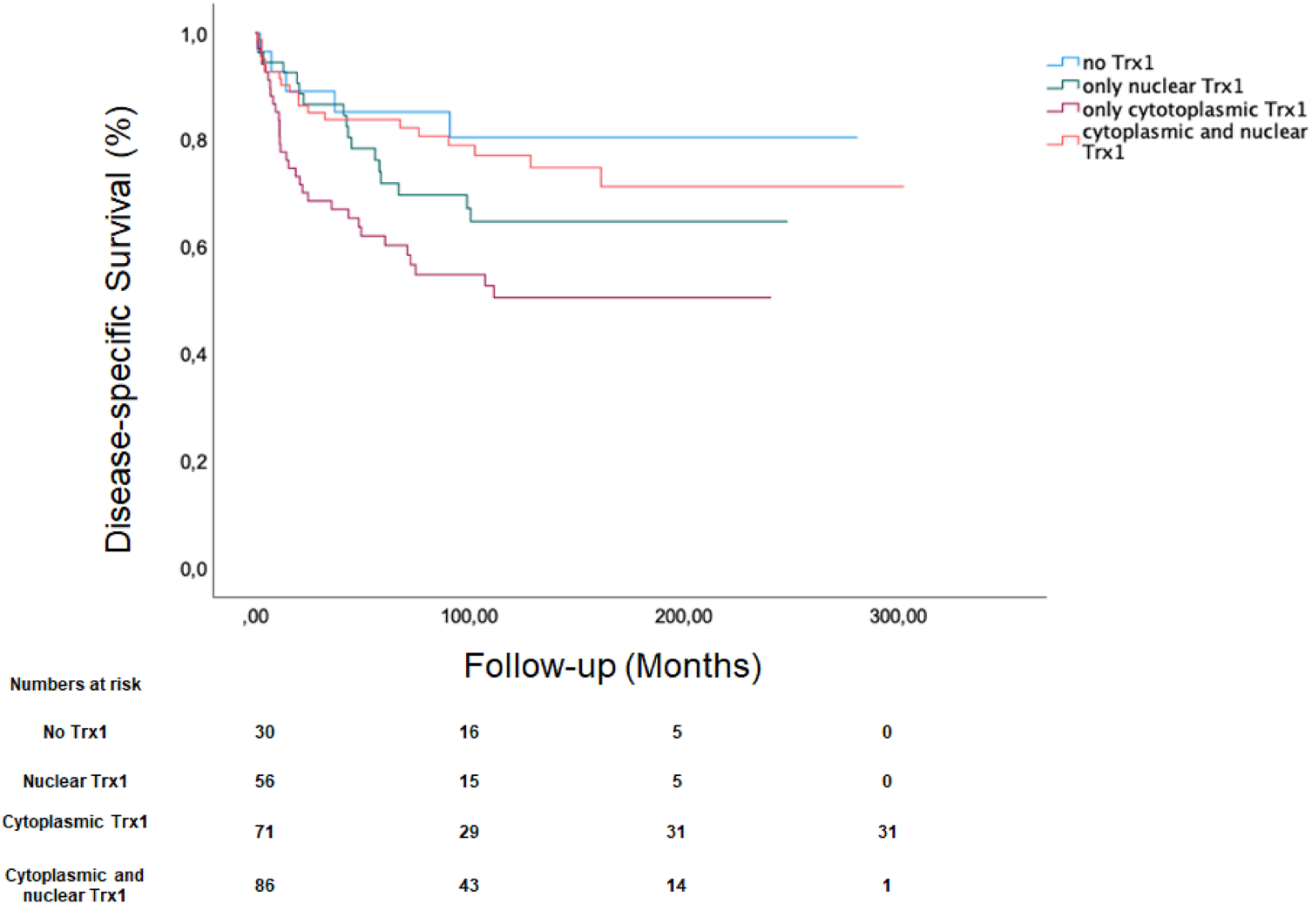
Compartment dependent Disease-specific survival estimation according to Trx1 Expression

The current analyzed in the TMA cohort consisted of 248 patients (171 men and 77 women) with a median age of 65 years (IQR 56.0–71.4) and a median follow-up time of 88.5 months (IQR 20.3–152.0 months). Seventy-two patients had died, because if ccRCC at the time of analysis. Patient and tumor characteristics of the whole TMA cohort is shown in supplementary Table 1 and the characteristics of the TCGA cohort is demonstrated in supplementary Table 2. There was a trend that any Trx1 vs. no Trx1 expression was associated with worse survival outcome HR: 2.02 95% CI 0.81–5. 02; *p* = 0.129. The comparison of associations of different compartments with DSS is demonstrated in Fig. 1. Tumors of patients who had exclusive expression of Trx1 in the cytoplasm had the worst survival outcome (HR, 95% confidence interval (CI): 3.13 (1.21–8.04)), Table 1). Any expression of Trx1 was considered in the different cellular compartments in this analysis.

**Table 1:** (A) Univariable association of compartment-specific Trx1 expression and cancer-specific survival; (B) Multivariable association of compartment-specific Trx1 expression, Stage, Size, Grade and Necrosis Score (SSIGN) with cancer-specific survival

A				
Variable	Hazard ratio	95% CI lower bound	95% CI upper bound	*p* value
No Trx1	Reference			
Nuclear Trx1	1.82	0.68	4.97	0.234
Cytoplasmic TrX1	3.13	1.21	8.04	0.018
Nuclear + Cytoplasmic TrX1	1.37	0.51	3.66	0.536

B				
Variable	Hazard ratio	95% CI lower bound	95% CI upper bound	*p* value
SSIGN 0–2 reference	–	–	–	–
SSIGN 3–4	1.43	0.61	3.03	0.407
SSIGN 5–6	2.77	1.13	6.18	0.013
SSIGN 7–9	4.97	1.86	10.35	< 0.001
SSIGN > 10	13.87	6.36	34.10	< 0.001
Trx1 continous	1.00	0.02	1.01	0.371

Next, the combined frequencies and intensities were investigated to better account for the protein levels as described above. For this purpose, we calculated a cutoff value of ≥ 10 using recursive partitioning. Patients with a combined score of ≥ 10 had better DSS than patients with a low combined score of < 10 (HR, 95% CI 0.62 (0.39–0.98)). In line with this result, tumors of patients with a high combined score demonstrated more often less advanced tumor features (Table 2). In multivariable analysis, high combined cytoplasmic Trx1 expression lost its significance for its association with survival outcome when adjusted for the prognostic groups of the SSIGN (Stage, Size, Grade and Necrosis) score. The multivariable analysis is demonstrated in Table 1B for Trx1 continuous expression. The investigation for categorized Trx1 showed similarly non-significant results.

**Table 2:** Association of Trx1 levels (frequencies x intensities) with clinicopathological features

Variable	Mean	SEM	*p* value
T1–T2	78.1	7.1	< 0.001
T3–T4	33.1	5.1	
N0	61.5	5.0	0.10
N1	23.8	14.9	
M0	64.2	5.4	0.018
M1	30.2	9.2	
G1	84.2	14.3	< 0.001
G2	64.0	6.0	
G3/G4	17.9	4.8	
Necrosis no	68.3	6.7	0.037
Necrosis yes	47.7	6.9	
Sarcomatoid no	63.2	5.1	0.008
Sarcomatoid yes	11.8	5.9	

### Results of the TCGA analysis

**Fig. 2:**
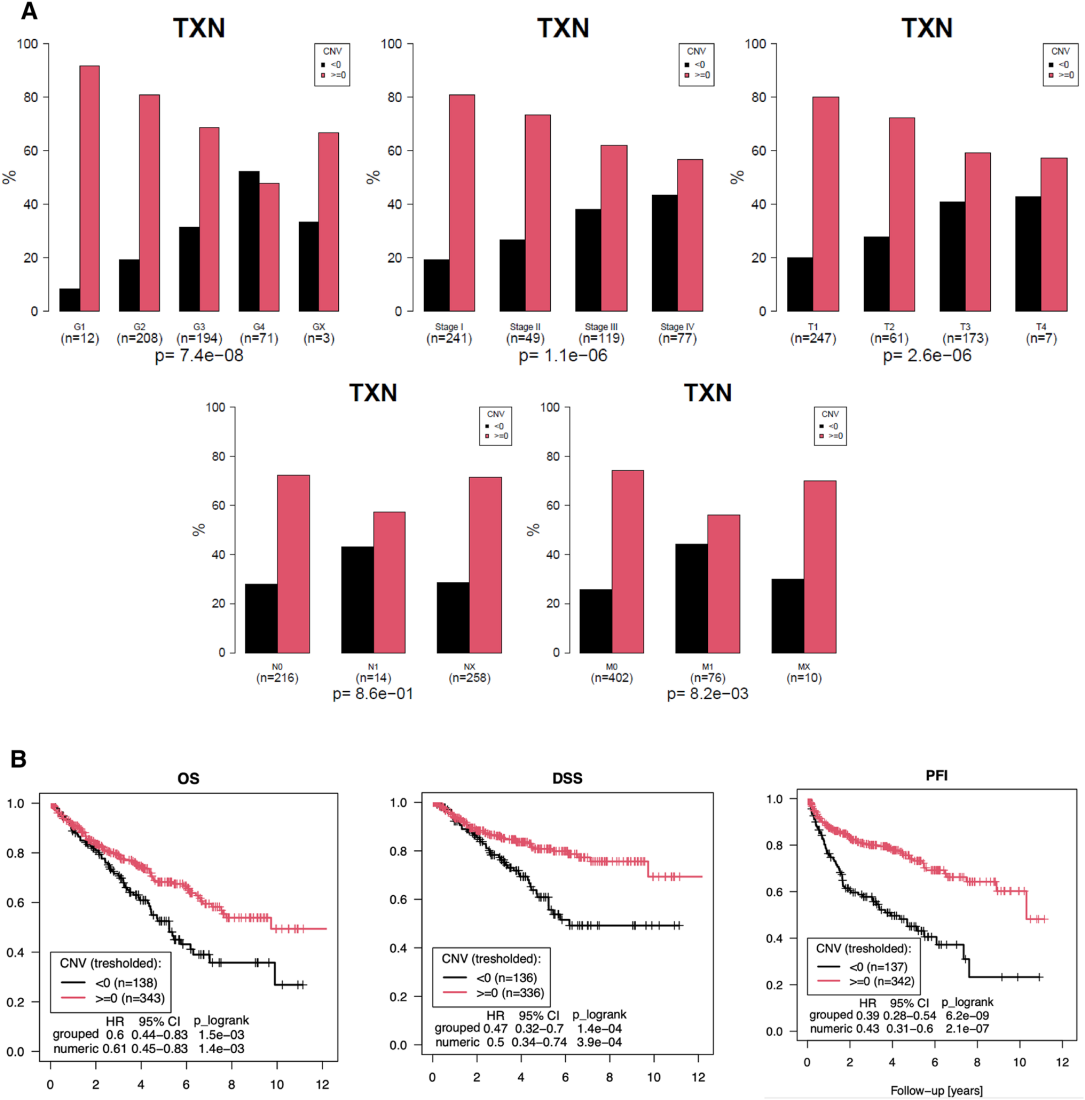
**A** Association Copy Number Variations (CNV) of thioredoxin 1 with clinicopathological features in The Cancer Genome Atlas project. Demonstrates the association of copy number variation with clinicopathological features. Black barrs (< 0) represent genome losses while red barrs (≥  0) represent diploid and gain variations in the genome. There was a clear correlation of genome losses with advanced clinicopathological features. **B** Overall and Cancer Specific Survival outcome according to copy number variations. Represents the association of copy number variations with overall survival, disease-specific survival and progression free intervall. Genome losses were associated with significantly worse survival outcome

The investigation of CNV of the Trx1 gene in the TCGA data revealed that diploid and gain genome variations in the Trx1 gene were significantly associated with less advanced tumor stages and clinicopathological features, while losses were more frequently observed in higher tumor stages and were associated with advanced clinicopathological features (Fig. 2A). Consequently, losses were associated with poor overall, disease specific, and progression free interval (Fig. 2B).

**Fig. 3:**
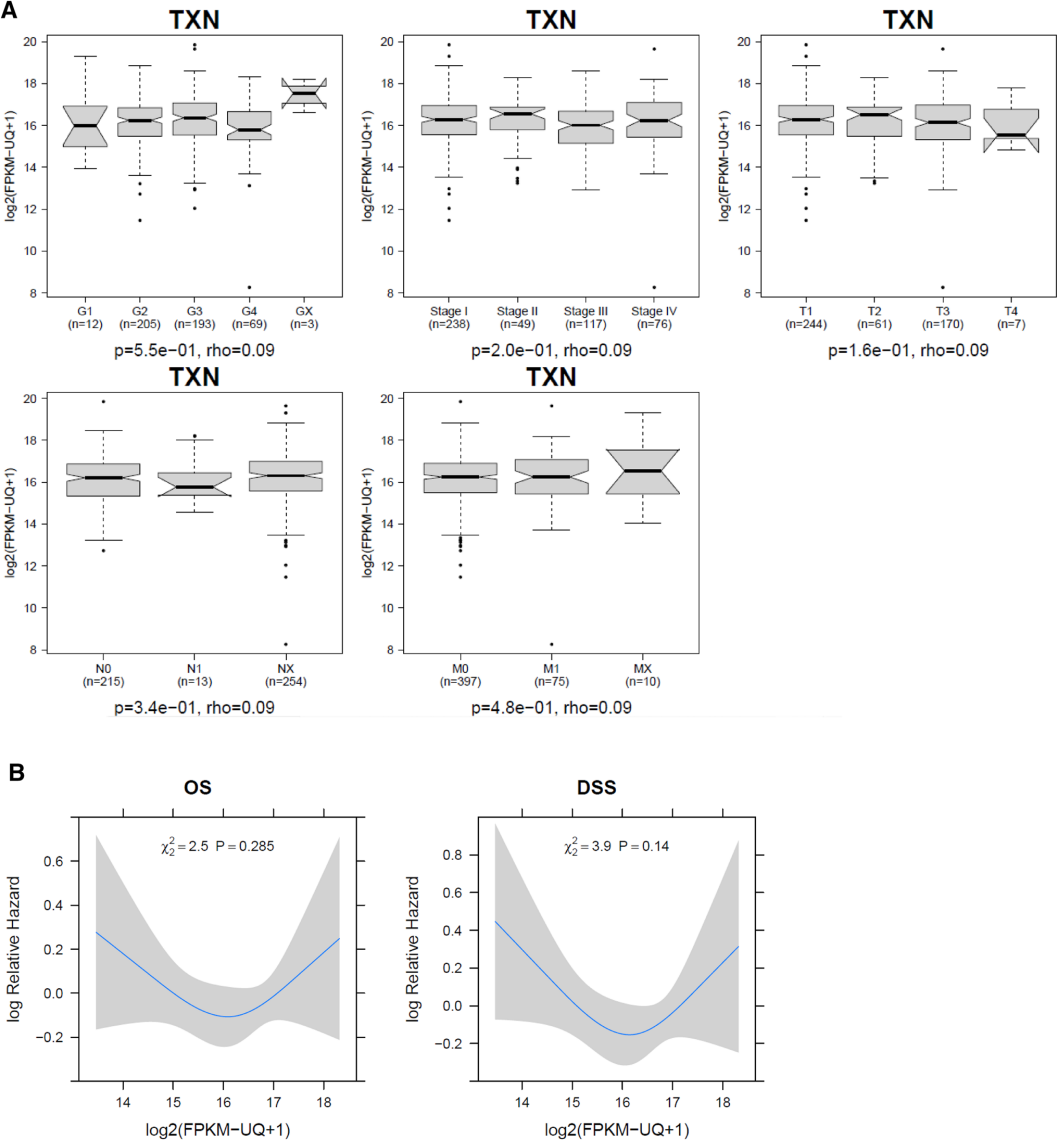
**A** Association of thioredoxin mRNA expression and clinicopathological features. Demonstrates the association of mRNA levels with clinicopathological features. There was no correlation of Trx1 mRNA levels with clinicopathological features. **B** Association of overall and cancer-specific survival with mRNA expression. Demonstrates the relative risk for death of any cause and disease-specific death in dependence of Trx1 mRNA levels. Patients with intermediate risk had the best survival outcome while patients with low and high expression had the worst survival outcome

Next, the gene expression levels were analyzed in the RNA seq data. There was no significant association of Trx1 mRNA levels with clinicopathological features (Fig. 3A) and there was also no continuous association of mRNA levels with survival outcome (Fig. 3B). Somatic mutations of Trx1 were only present in two patients.

## Discussion

The current study aimed to explore a possible role of the most prominent member of the thioredoxin family, Trx1, in tumor biology of ccRCC. We demonstrate that there is a compartment-specific association of Trx1 expression with survival outcome in ccRCC. Additionally, the amount of Trx1 protein levels was correlated with clinicopathological features: lower Trx1 protein levels were associated with advance clinicopathological features. Furthermore, there was a correlation of CNV (losses) with advanced tumor stages, while there was no clear correlation for Trx1 mRNA levels with either clinicopathological features or survival outcome.

Redox signaling is a compartment-specific biological process. Some of the thioredoxins are compartment-specific proteins and others are able to shuttle between cellular compartments [1]. The current study shows that expression of Trx1 in the cytoplasm is associated with poor prognosis in ccRCC but on the other hand higher levels of Trx1 were associated with less advanced clinicopathological features. This finding underscores previously published reports of some of our group members. Berndt et al*.* showed that Trx1 suppresses the translation of HIF-2α and HIF-2α is a major tumor progressor in ccRCC [7, 8]. This may be more pronounced if HIF-2α is located in the cytoplasm [13]. These pre-clinical data have been underscored in clinical trials that investigated small molecule agents inhibiting HIF-2α in patients with metastatic ccRCC [14]. First data of clinical phase I and II studies have demonstrated that these agents are active in patients with ccRCC [15]. In context with the findings of the current study, the less advanced clinicopathological features in tumors with higher Trx1 expression may be an effect of Trx1 on HIF-2α expression. ccRCCs have a specific tumor biology. In physiological conditions with sufficient oxygen supply, HIF-2α is constantly degraded via a process that is orchestrated by the *von Hippel Lindau* protein (pVHL). However, in roughly 90% of ccRCC, pVHL is inactivated and thus HIF-2α is overexpressed even under normoxic conditions [16]. The particular redox regulation via Trx1 in cells with inactive or missing pVHL has yet to be determined. These investigations have to take the compartment-specific differences particularly into account.

The investigations of genetic aspects in the TCGA data have demonstrated that there is a clear correlation between CNV, advanced clinicopathological features and poor survival outcome of ccRCC patients. We expected that copy number losses may be linked to lower RNA expression and that lower RNA expression is linked to poor prognostic tumor features and prognosis. However, our analysis of the RNASeq data from the TCGA was unable to demonstrate an association of RNA levels with poor prognostic clinicopathological features. In fact, cubic spline analyses demonstrated that those patients with ccRCCs with medium RNA expression of the Trx1 gene had the worst survival outcome. Although, CNVs are known for a long time and the prognostic significance of CNVs has been demonstrated in several studies in ccRCC, the biological relevance is still not well characterized [17, 18]. Our finding underscores that CNVs and RNA expression are not necessarily linked and, therefore, the biological basis for the association of genomic CNV losses of Trx1 and advanced clinicopathological features needs to be subject of future additional investigations.

The current findings have several limitations that need to be considered when interpreting these results. This is a retrospective study. The composition of the study could have been more balanced in terms TNM stages. Additionally, the follow-up could have been longer.

Some of our findings seem to be contradictory, e.g. higher Trx1 levels in the cytoplasm are associated with worse survival outcome while higher Trx1 levels are per se associated with less advanced clinicopathological features. Redox signaling is a process that is compartment and context specific like other biological processes e.g. the tumor suppressive role of p53 or the transcriptional regulation of NFκB. We have interpreted our current results in line with this biological knowledge. These prima vista contradictory results leave room for additional investigations, particularly the regulative role of Trx1 on HIF-2α is an attractive target with respect to the current phase III clinical trials that investigate HIF-2α inhibition as a therapeutic concept in ccRCC and other cancer types. Our group has a major interest in this field and additional investigations are currently underway.

In summary, the current study suggest that Trx1 has a role in tumor biology of ccRCC. Possible explanations for an important role of redox signaling ccRCC are regulation of immune response, metabolic pathway regulations and the regulation of HIF-2α expression. Thus, the current study strongly advises intensified investigations of redox signaling pathways in ccRCC.

## Supplementary Information

Below is the link to the electronic supplementary material.

Supplementary file1 (DOCX 29 kb)

## Data Availability

Data are mentioned in the manuscript are freely available in the TCGA found at http://cancergenome.nih.gov or will be provided upon request from the corresponding author.
