# Supplementary material for: Thioredoxin 1 (Trx1) is associated with poor prognosis in clear cell renal cell carcinoma (ccRCC): an example for the crucial role of redox signaling in ccRCC
Source: World J Urol. 2021 Dec 2;40(3):739–46. doi: 10.1007/s00345-021-03900-5 (PMC8948103; doi:10.1007/s00345-021-03900-5)
Supplement: Supplementary file 1 — Supplementary file1 (DOCX 29 kb) [file 345_2021_3900_MOESM1_ESM.docx]

Supplementary Table 1

| **Clinicopathological parameters of the TMA cohort analyzed for Trx1.** | | |
| --- | --- | --- |
| **Feature** | **Parameter** | **Patients** |
| **Age** | **Median (IQR)** | 65.3 (56.0 – 71.4) |
| **Follow-up (months)** | **Median (IQR)** | 88.5 (20.8 – 152.0) |
| **Gender (%)** | **Male**  **Female** | 171 (69)  77 (31) |
| **pT-Stage (%)** | **T1a**  **T1b**  **T2**  **T3a**  **T3b**  **T3c**  **T4** | 89 (36)  57 (23)  0 (0)  93 (37)  7 (3)  6 (1)  0 (0) |
| **p/cN-Stage (%)** | **N0**  **N1** | 237 (95)  12 (5) |
| **cM-Stage (%)** | **M0**  **M1** | 216 (87)  33 (13) |
| **Fuhrman Grading (%)** | **G1**  **G2**  **G3**  **G4** | 38 (15)  171 (69)  40 (16)  0 (0) |
| **L-Stage** | **L0**  **L1** | 233 (94)  16 (6) |
| **V-Stage** | **V0**  **V1**  **V2** | 170 (68)  77 (31)  2 (1) |
| **Sarcomatoid Features (%)** | **No**  **Yes** | 232 (93)  17 (7) |
| **Necrosis** | **No**  **Yes** | 147 (59)  102 (41) |

**Supplementary Table 2.**

**Clinicopathological features of the TCGA cohort:**

| **Characteristics of patients/primary tumors** | **levels/summary statistics** | **n=459 KIRC tumors** | |
| --- | --- | --- | --- |
|  |  | **No.** | **%** |
| **Sex** | male | 293 | 63.8 |
|  | female | 166 | 36.2 |
| **Age (years) at diagnosis of primary RCC** | median (range) | 61 (29-90) | |
| **T** | 1 | 230 | 50.1 |
|  | 2 | 56 | 12.2 |
|  | 3 | 168 | 36.6 |
|  | 4 | 5 | 1.1 |
| **N** | 0 | 205 | 44.7 |
|  | 1 | 12 | 2.6 |
|  | X | 242 | 52.7 |
| **M** | 0 | 364 | 79.3 |
|  | 1 | 68 | 14.8 |
|  | X | 27 | 5.9 |
| **G^a^** | 1 | 10 | 2.2 |
|  | 2 | 192 | 41.8 |
|  | 3 | 186 | 40.5 |
|  | 4 | 68 | 14.8 |
|  | X | 1 | 0.2 |
| **Stage^a^** | I | 224 | 48.8 |
|  | II | 44 | 9.6 |
|  | III | 118 | 25.7 |
|  | IV | 71 | 15.5 |
| **Follow-up time (years)** | median (range) | 3.6 (0-12.4) | |
| **Vital status** | alive | 310 | 67.5 |
|  | dead | 149 | 32.5 |
| **Cancer-related death^a^** | no | 360 | 78.4 |
|  | yes | 89 | 19.4 |
| **Progression‐free-intervall** | no | 321 | 70 |
|  | yes | 138 | 30 |
| ^a^Percentages do not sum up to 100% due to missing values. | |  |  |

**Clinical characteristics of the analyzed TCGA KIRC cohort (n=459). In this analysis, patients with none-ccRCC and neoadjuvant treatment have been excluded according to Chen et al. (Chen, F., Zhang, Y., Senbabaoglu, Y. et al.: Multilevel Genomics-Based Taxonomy of Renal Cell Carcinoma. Cell Rep, 14: 2476, 2016). Available data sets have been different for the individual analyses.**

# Supplements: Description of Methods and Materials.

## Tissue Micro Array

Core tissue biopsies (0.6 mm in diameter) were taken from selected morphologically representative regions of paraffin-embedded renal tumors and precisely arrayed using a custom-built instrument. Each block was evaluated from an experienced pathologist before it was included in the study. Then 4 µm thick sections of the resulting tumor tissue microarray block were transferred to glass slides. The process has been described previously in detail [9].
Immunohistochemistry: For antigen retrieval, a citrate buffer of pH 9.0 was used. Slides were stained with the primary antibody for Trx1 with a concentration of 1:200 and incubated overnight. Endogenous peroxidase was cleared with 1 % hydrogen peroxide, and positive reactivity of primary antibodies was performed by the HRP polymer and DAB as the chromogen substrate (Dako, Glostrup, Denmark).

## Antibody Construction

The process of Trx 1 antibody construction has been described previously in [11]. TRX1 was produced in rabbits by injection of 40 µg recombinant protein in 100 µl phosphate buffered saline (PBS; PAA Laboratories, Cölbe, Germany) mixed freshly with 500 µl of complete Freund's adjuvant (Sigma, St. Louis, USA). Every 4 weeks, subsequent injections were performed with 40 µg of the antigens diluted in incomplete Freund's adjuvant (Sigma). Blood was collected following the third to sixth injection and the serum was heat-inactivated at 56 °C for 20 min. All sera and purified antibodies produced for this study were validated for specificity by Western blotting and immunohistochemistry comparing antibody-stained samples with samples stained with antibodies pre-incubated with 10–200 µg/ml of the specific antigen (see [11]). In case of precipitation of the antigen–antibody complexes during control experiments, antigens were coupled to CNBr activated sepharose. Antigen-specific antibodies were subsequently removed by chromatography. Only antibody preparations that exhibited exclusive antigen-specific staining were used.
